# Ranking Factors Affecting the Attraction of Foreign Medical Tourists in Hospitals Affiliated to Mashhad University of Medical Sciences Based on Marketing Mix Model

**Published:** 2018-10

**Authors:** Reza AZIMI, Ghahraman MAHMOUDI, Habib-Allah ESMAEILI

**Affiliations:** 1. Health Care Management, Sari Branch, Islamic Azad University, Sari, Iran; 2. Hospital Administration Research Center, Sari Branch, Islamic Azad University, Sari, Iran; 3. Dept. of Biologic Statistics, Faculty of Health, Mashhad University of Medical Sciences, Mashhad, Iran

**Keywords:** Ranking, Medical tourism, Mixed marketing, Hospitals, Iran

## Abstract

**Background:**

This study was designed to rank factors affecting attraction of foreign medical tourists based on marketing mix model.

**Methods:**

In this descriptive study, data were collected through a questionnaire. Foreign patients, hospitalized from Jan 2015 to Sep 2016 in all hospitals of Mashhad were chosen as the study population (13 hospitals). Data analysis was conducted using Friedman test and Wilcoxon test in SPSS 21 at a significance level of 0. 05.

**Results:**

Factors of staff, service and process gained the highest score from the perspective of patients; while price, facilities and promotion scored the lowest among parameters affecting the attraction of medical tourists to hospitals of Mashhad. In this context, factors of staff (96%) and price (66%) had the highest and lowest effect on absorption of tourists, respectively.

**Conclusion:**

To promote medical tourism, important factors such as staff, service, and process should be further stressed and variables of price, facilities and promotion demand for a revision by the authorities in this industry.

## Introduction

Tourism is predicted to become the most profitable industry of the world by 2020. Among diverse types of tourism, medical tourism is regarded as one of the key areas of tourism industry in the world, which has been on rise in recent years. The revenue generated by each medical tourist is three times as high as revenue brought by ordinary tourists (1).

Considering the tremendous financial turnover of this industry in the world, today many developing countries in Asia, Latin America Africa are competing for the absorption of medical tourists. Of course, since each of these countries offers medical services in a specific area, which serves as their competitive edge, it is difficult to determine the most popular medical tourist destination. Overall, major medical tourism destinations in the world are Costa Rica, Mexico, the US, Singapore, Thailand, Malaysia, India, Philippines, Taiwan, South Korea and Turkey(2).

The potentials of medical tourist absorption in international market depend on standard international quality criteria. However, Iran still faces great challenges in exploiting potentials in this field, the most important of which is inconsistency of national health facilities and international standards. In other words, among 4 factors of price, quality, availability of services and time, which are recognized as key factors essential for medical tourism growth, quality is of paramount importance(3). A more accurate estimation requires the inclusion of other factors like place, staffs, processes, promotion, and facilities. Specialty and subspecialty medical centers, low-cost services, natural attractions, proximity to Arabic markets and cultural and linguistic similarity with neighboring countries are some features that facilitate the absorption of foreign patients and medical tourists to Iran(4). Through recognition and reasonable utilization of capabilities and potentials of medical tourism, Iran can emerge as the hub of medical tourism in Asia. However, in the absence of an integrated and meticulous national plan, these opportunities have not been fully tapped. In addition, medical tourism development and effective management require some basic groundwork. The experiences of other countries suggest that long-term planning in tourism can contribute to market development without encountering any serious challenges (5).

Medical tourism in Iran has great potentials for job creations and can be construed as an important source of exchange. Thus, greater attention has to be paid to factors affecting the attraction of medical tourists. Some studies have addressed this issue, but few researchers have analyzed these factors from the perspective of patients. Therefore, this study seeks to identify and rank factors influencing the attraction of foreign medical tourists in hospitals affiliated with Mash-had University of Medical Sciences using marketing mix model.

## Methods

As an applied research with a descriptive design, this research is based on cross-sectional analysis. The study population consisted of all foreign patients admitted to hospitals affiliated to Mashhad University of Medical Sciences, Mashhad, Iran in 2016. Overall, there are more than 47 hospitals associated with the Mashhad University of Medical Sciences, out of which 33 are in the city of Mashhad, Iran. Thirteen hospitals including Army 550, Aria, Imam Reza, Imam Zaman, Imam Hadi, Shahid Kamyab, Bentolhoda, Jawad al-Aemme, Razavi, Qaem, Mehr, Mehregan, and Musa ibn Ja’far were incorporated in this study. Convenience sampling method was used for selecting participants.

Given the huge variance of price variable compared to other variables, a sample size of n=127 was estimated in the preliminary study, with a price variance of 828, accuracy of 95% and a 5-unit precision. Finally, a sample of n=136 was considered for the study.

Given the ethnic diversity of medical tourists, first, the list of patients admitted to hospitals receptive of medical tourists was obtained. As it turned out, most patients were from neighboring Arab countries in the Persian Gulf. The standard questionnaire was adapted from a research conducted in Shiraz University of Medical Science (6), translated into Arabic. The content validity of the questionnaire was verified by 5 professors and their ideas were included. To measure the reliability, questionnaires were filled out by patients with an at least one-day interval and the correlation was calculated (r = 0. 88.)

The questionnaire had three parts. The first part consisted of 8 items that addressed demographic data of participants. The second part contained 6 items on how patients had learned about the hospital, what mode of transport they had used to commute to the hospital, their length of stay, reason of admission and type of surgery. The third part consisted of 7 elements of marketing mix: services (11 items), place (3 items), promotion (13 items), price (9 items), people (12 items), process (6 items) and facilities (19 items). The items in the third part were scored on a three-point scale (Yes, No, to some extent) which were assigned 0 to 2 values respectively. The third part contained a total of 73 items.

After confirming the validity and reliability of the questionnaire, they were delivered to select hospitals of Mashhad Medical University to be distributed among Arab tourists by the hospital liaison, who was one of the hospital staff and preferably familiar with Arabic or English languages. Prior to the study, liaisons were briefed on research objectives and questionnaires and a Persian and English version of questionnaire were submitted to each questioner so that they could address possible questions of patients. The questionnaire took about 20 min to be completed, provided that participants were able to do it without interruption. Due to the special condition of patients, they were often obliged to fill out the questionnaire in multiple stages. Questioning was started in Jan 2015 and lasted until Sep 2016. Friedman test was used to rank parts of the questionnaire. The data analysis was performed by SPSS 21(Chicago, IL, USA) at a significance level of 0. 05.

## Results

Overall, 136 non-Iranian patients who were willing to participate were included. The total score of items in healthcare marketing mix questionnaire (service, place, promotion, price, people, process, facilities) was calculated by summing up scores of each respondent. Based on the defined rating system, they were assigned to three groups (Yes, No and to some extent).

The highest mean belonged to factors of people (5. 58) followed by services (4. 38), process (4. 28), place (3. 83), promotion (3. 56), and facilities (3. 44) and price (2. 93) respectively (Table 1).

**Table 1:** Mean and standard deviation of the questionnaire

***Factor***	***Mean ± SD***	***Mean Rating***
Service	1. 53± 0. 430	4. 38
Place	1/48. ± 0/42	3. 83
Promotion	1/37. ± 0/48	3. 56
Price	1/25. ± 0/50	2. 93
People	1/73. ± 0/32	5. 58
Process	1/49. ± 0/49	4. 28
Facilities	1/36. ± 0/47	3. 44
Friedman	X^2^=141/7	*P*<0. 001

To compare the impact of factors involved in attracting foreign medical tourists, Friedman test was employed. Then, Wilcoxon test with bone-ferny correction was used to provide a pairwise comparison of questionnaire factors (Table 2). A significant difference was revealed between service and other factors, except for place and process factors. Moreover, place was found to significantly difference from other factors, with the exception of promotion and process. With regard to promotion, it was significantly differenced from all other factors, except for facilities. There was a significant difference between price, people, and processes and other factors.

**Table 2:** Pairwise comparison of factors affecting the attraction of foreign medical tourists

***Group***	***Wilcoxon test***	***Probability***
Service^*^ place	−1/64	0. 144
Place ^*^ promotion	−4/68	< 0. 001
Service ^*^ price	−5/98	< 0. 001
Service ^*^ People	−5/43	< 0. 001
Service ^*^ Process	−0/89	0. 372
Service ^*^ Facilities	−4/10	< 0. 001
Place^*^ promotion	−1/23	0. 216
Place^*^price	−3/81	< 0. 001
Place ^*^ people	−5/53	< 0. 001
Place^*^ process	−1/19	0. 232
Place^*^ facilities	−1/82	0. 067
Promotion^*^ price	−3/37	< 0. 001
Promotion ^*^ staff	−7/66	< 0. 001
Promotion^*^ process	−3/20	< 0. 001
promotion ^*^ facilities	0/45	0. 649
Price ^*^ people	−8/95	< 0. 001
Price ^*^ process	−5/23	< 0. 001
Price ^*^ facility	−2/36	0. 018
People ^*^ process	−6/49	< 0. 001
People ^*^ facilities	−8/29	< 0. 001
People^*^ facilities	−3/99	< 0. 001

## Discussion

The results of study revealed that all seven factors of marketing mix -service- place- promotion- price- people- processes and facilitates – affected the attraction of foreign medical tourists. However, some disparities with previous studied were observed.

One underlying reason for the development of medical tourism was the availability of low-cost health services at the destination (7). Provision of services at reasonable price together with tourism facilities was highly important (8). Cost of care had a significant positive effect on the motivation of foreign patients to seek medical services in Iran (9). However, in a recent study, the factor of price was found to have less impact on attracting medical tourists, which is inconsistent with other studies.

Primarily the quality and standards of medical supplies were stressed at the cost of devaluing the price factor (2). Although low price was one of the competitive edges of tourism destinations in Asia, exchange rate fluctuations had undermined this advantage (10).

The process of attracting tourists is a key factor influencing the growth of medical tourists. Weak marketing and promotion system regarding the admission and treatment procedures in the city of Mashhad, inefficient electronic admission system and payments methods as well as weak financial transactions systems in hospitals have influenced the attraction of medical tourists (11). The above study failed to draw a comparison between process and other factors. While our results indicated that patients were satisfied with the process of treatment, this factor was ranked third after service and people factors.

Many researchers focused on promotion factor, with their findings reflect the importance of this factor in attracting foreign tourists. Marketing plays a pivotal role in maintaining competition. Therefore, it is vital to understand the attitude and behavior of medical tourists to support government agencies and stakeholders with the aim of facilitating and regulating tourism policy. This helps establishes a balance between customer expectations and their information (12). Contrary to the belief of most researchers and journalists, who refer to the low cost of treatment in the destination countries as the main motivating factor of patients, advertising campaigns aimed at attraction of tourists are often geared towards international and national reputation and promotion of a wide range of specialized medical procedures offered to international patients (13). Both of these studies demonstrate the importance of promoting, which took precedence over the price factor. These results are in agreement with our study.

A review of literature reveals a general consensus among researchers about the important role of quality and quantity of products in attracting customers. Today, all providers tend to focus on distinguishing features of their product, particularly due to the attention of consumers to such distinctions. The overall medical and service quality were more important than all other features (14). Unlike other studies that introduced low cost of medical services as the main drive for medical tourists. Product quality was one of the determinants of customer attraction, which was priori-tized over the price of medical services. What can be gathered from views of researchers is that product quality is the cornerstone of customer attraction. Therefore, special attention should be paid to quality and distinguish features of products as key factors in drawing medical tourists (2). According to results of this study, it is as the second important factor in attracting developing tourism.

Facilities and equipment would be of little use without expert and experienced staff. Availability of motivated and skilled workforces is pivotal to the success and progress of an enterprise and its customer attraction. Becoming a successful medical tourism destination requires various skilled employees along with quality products and services (15). Trust in Iranian doctors is one of the factors contributing to the development of medical tourism (4). Providing in-service training courses at national and international level to improve scientific skills of professional workforce and recruitment of physicians with board of specialty and training human resources in the health sector with the aim of promoting the quality of services can help draw medical tourists (16). Consistent with the results of this study, staff is a key factor in absorption of medical tourists.

Lack of appropriate leisure/entertainment facilities and inefficient transportation system are other issues hampering the attraction of medical tourists to Iran (17). The unsatisfactory condition of Shiraz hospitals in this regards (18). The present study, however, does not show such a situation in hospitals of Mashhad. More than anything, medical tourists seek to receive medical services, but they also need amenities before, during, and after treatment, which is complementary to therapy services. However, the effect of facilities factor was higher than price with other factors being of less importance.

## Conclusion

In the view of patients, factors of staff, service and process have the greatest effect on attracting medical tourists to hospitals affiliated to Mashhad University of Medical Sciences with the lowest satisfaction belonging to factors of price, facilitates and promotion. Putting these results into perspective, it is necessary to pay further attention to the adoption of appropriate strategies to recruit skilled people, provide services based on international standards and change processes in hospitals affiliated to Mashhad University of Medical Sciences. Moreover, to promote competition, new advertising media such as websites and mass media should be adopted and special attention should be paid to the revision of fees.

## Ethical considerations

Ethical issues (Including plagiarism, informed consent, misconduct, data fabrication and/or falsification, double publication and/or submission, redundancy, etc.) have been completely observed by the authors.
